# Targeting revascularization via tryptophan-indole-NETs axis: the synergistic power of acupuncture and rt-PA thrombolysis in ischemic stroke

**DOI:** 10.3389/fneur.2025.1596158

**Published:** 2025-07-04

**Authors:** Siqi Chang, Yi Zhao, Chengfeng Li, Xinyu Liu, Zixuan Fang, Zihao Peng, Mingliang Gao, Yawen Xia, Guangxia Ni

**Affiliations:** ^1^College of Acupuncture-Moxibustion and Tuina, Nanjing University of Chinese Medicine, Nanjing, China; ^2^Key Laboratory of Acupuncture and Medicine Research of Ministry of Education, Nanjing University of Chinese Medicine, Nanjing, China; ^3^Jiangsu Joint International Research Laboratory of Chinese Medicine and Regenerative Medicine, Nanjing University of Chinese Medicine, Nanjing, China

**Keywords:** ischemic stroke, thrombolysis, acupuncture, tryptophan metabolism, neutrophil extracellular traps, vascular function

## Abstract

**Background:**

The Xingnao kaiqiao (XNKQ) method, meaning “awakening the mind,” is an acupuncture technique developed by Academician Xuemin Shi. It has shown promise in ischemic stroke management. This study aims to explore the synergistic power and underlying mechanisms of XNKQ method and recombinant tissue plasminogen activator (rt-PA).

**Methods:**

A rat embolic stroke model was utilized to replicate the pathological processes of ischemic stroke. The effects on cerebral perfusion and vascular leakage were assessed *in vivo* and *in vitro*. Both non-targeted and tryptophan-targeted metabolomic analyses were conducted to identify differential metabolites. Additionally, neutrophil extracellular traps (NETs) markers and vascular leakage were detected to elucidate the crosstalk.

**Results:**

The combination of acupuncture and rt-PA has been shown to significantly ameliorate middle cerebral artery occlusion (MCAO)-induced cerebral infarction, blood flow obstruction, neurological deficits, and histopathological damage in rat models. Acupuncture may also reduce hemorrhagic transformation and cerebral edema linked to ultra-time window rt-PA thrombolysis. Metabolomic studies suggest acupuncture enhances the efficacy and safety of rt-PA via the tryptophan-indole metabolic pathway, facilitating the degradation of NETs proteases and reducing inflammation. Furthermore, NETs accumulation is linked to increased brain vascular permeability as evidenced by NETs regulators (LPS or DNase I). Acupuncture and NETs inhibitors both offer similar vascular protection during ultra-time window rt-PA thrombolysis, with acupuncture being more effective in reducing cerebral infarction.

**Conclusion:**

The degradation of NETs via the tryptophan-indole pathway may facilitate the restoration of vascular structure and function following ischemic stroke. Acupuncture has the potential to modulate this pathway, thereby possibly extending the therapeutic window for rt-PA thrombolysis.

## 1 Introduction

Ischemic stroke represents a major contributor to mortality and disability among adults worldwide (1). Clinically, recombinant tissue plasminogen activator (rt-PA) is regarded as the gold standard treatment for ischemic stroke, provided it is administered within 4.5 h of symptom onset. Intravenous rt-PA effectively facilitates the breakdown of fibrin, thereby rapidly restoring cerebral blood flow to the ischemic penumbra and mitigating neurological deficits (2). Nevertheless, only approximately 5–7% of ischemic stroke patients receive intravenous rt-PA therapy, a limitation attributable to both the stringent therapeutic window and the associated risk of severe complications, most notably intracerebral hemorrhage (ICH) (3). Consequently, improving the safety of thrombolytic therapy, particularly beyond the established time constraints, represents a critical unmet need in contemporary ischemic stroke management.

Vascular structural dysfunction plays a critical role in the pathogenesis and progression of cerebral infarction and imposes significant limitations on thrombolytic therapy (4). This dysfunction compromises vascular drainage capacity, precipitates focal occlusions, and exacerbates infarct expansion, thereby impeding drug distribution and diminishing the therapeutic efficacy of rt-PA thrombolytic therapy. It leads to impaired vasodilatory function, exacerbates ischemia–reperfusion injury during thrombolysis, increases vascular permeability, and ultimately elevates the risk of ICH and cerebral edema (5). Therefore, understanding the mechanisms underlying vascular injury following ischemic insult or thrombolytic intervention could yield substantial progress in the prevention and treatment of cerebral infarction.

Fu-Dong Shi and Sophie Gautier have emphasized the critical role of the inflammatory response in maintaining vascular function after cerebral infarction, demonstrating its dual role in both vascular protection and injury exacerbation (6, 7). Neutrophils, being the predominant immune cell population and a pivotal effector of innate immunity, deserve particular attention in this context (8, 9). During the initial phase of cerebral ischemia and hypoxia, neutrophils are activated by the substantial secretion of cytokines, including tumor necrosis factor (TNF), interleukin-1β (IL-1β), and interleukin-8 (IL-8). This activation results in the production of a significant amount of reactive oxygen species (ROS), promotes the release of neutrophil extracellular traps (NETs), facilitates the phagocytosis of harmful substances, and facilitates the phagocytosis of harmful substances, and assists in the removal of microvascular necrosis (10, 11). However, excessive activation of neutrophils and overproduction of NETs can exacerbate secondary vascular inflammatory injury. In models of tumors and sepsis, NET-associated proteases have been demonstrated to disrupt endothelial cytoskeletal architecture, culminating in microvascular hyperpermeability and barrier impairment (12, 13). Collectively, NET formation may contribute to vascular dysfunction. Moreover, emerging evidence indicates that rt-PA markedly augments neutrophil infiltration into ischemic regions, and an elevated neutrophil-to-lymphocyte ratio (NLR) correlates with adverse clinical outcomes (14, 15). Based on these observations, we hypothesize that the inhibition of NETs may represent a critical target for the prevention and treatment of cerebral infarction and thrombolytic complications.

Academician Xuemin Shi pioneered the Xingnao Kaiqiao (XNKQ) method, which has demonstrated significant efficacy in treating stroke, as substantiated by large-scale clinical observations (16). In a preliminary clinical study conducted by our research group, we observed that timely intervention with XNKQ method, can enhance the effectiveness of intravenous thrombolysis with rt-PA in patients suffering from ischemic stroke (17, 18). This approach also appears to reduce the incidence of symptomatic ICH. Notably, our experimental studies revealed a novel finding: acupuncture combined with ultra-extended window rt-PA thrombolysis maintains therapeutic safety and efficacy for ischemic stroke treatment (19). This finding could potentially expand the eligibility for thrombolytic therapy to a broader patient population in clinical settings. Nonetheless, the therapeutic mechanisms underlying this approach require further elucidation. The present article systematically examines the acupuncture-thrombolysis synergy and its molecular underpinnings.

## 2 Materials and methods

### 2.1 Animals

Adult male SD rats (300 ± 20 g) and maintained at SPF level, were obtained from Shanghai Weitonglihua Animal Co., Ltd. (Shanghai, China; license no. SCXK (Hu): 2018-0006). The rats were housed in a controlled environment with regulated temperature and humidity, following a 12 h light/dark cycle. This research received approval from the Institutional Animal Care and Use Committee at Nanjing University of Chinese Medicine, and all experimental procedures were carried out in strict adherence to the guidelines set forth by the National Institutes of Health Animal Care and Use Committee. The experiments conducted in this research adhered to the ARRIVE guidelines (202408A020).

### 2.2 Animal grouping

The rats were systematically allocated into various groups, which included Sham, Model, rt-PA (4.5 h, 6 h), A + 6 h rtPA, NA + 6 h rtPA, LPS + 6hrt PA, DNase I + 6hrtPA utilizing a randomization technique. The model group represents the ischaemic stroke state without any treatment. Additionally, a Sham group was created to account for the influence of non-pathological variables related to the surgical procedure. This cohort did not undergo the administration of a thromboembolus into the middle cerebral artery following the dissection of the carotid artery.

### 2.3 Establishment of the embolic stroke model

First, blood is collected from the inner canthus of the donor rat using a capillary glass tube containing thrombin (1,000 U/mL) with an inner diameter of 0.5 mm. After 24 h the clot was removed and cut it into small pieces, a modified PE-50 catheter, which was connected to a syringe, was utilized to inject the clot into the middle cerebral artery (MCA). Anesthetized rats were immobilized, and their skin was disinfected prior to the exposure of the cervical blood vessels. The external carotid artery (ECA) was subjected to ligation and temporary clamping, accompanied by a partial arteriotomy. The modified PE-50 catheter, which contained the blood clot, was advanced into the ECA towards the internal carotid artery (ICA) until it reached the origin of the MCA. After a slight retraction, the clot was gradually infused with a saline solution. The catheter was subsequently removed after a duration of five minutes. The effectiveness of the model was validated by confirming the obstruction of cerebral blood flow, which was monitored using a laser speckle imaging system (RWD, Shenzhen, China). Successful obstruction was indicated by a significant decrease in cerebral perfusion.

### 2.4 Acupuncture treatment

Rats assigned to the acupuncture group underwent treatment at the Shuigou (GV26) and bilateral Neiguan (PC6) acupoints simultaneously with thrombolytic therapy. The selection of acupoints was guided by the acupoint map for experimental animals as published by the Chinese Acupuncture Society. The GV26 acupoint is situated at the junction of the upper one-third and middle one-third of the upper lip, whereas the PC6 acupoint is positioned approximately 3 mm proximal to the wrist crease. Stainless steel acupuncture needles, possessing an outer diameter of 0.3 mm, were inserted to a depth of 2–3 mm at both the GV26 and PC6 acupuncture points. The acupuncture procedure involved techniques of lifting, thrusting, twirling, and reducing, each applied for one minute, after which the needles were retained for a duration of 30 min.

### 2.5 Thrombolysis treatment

Rats assigned to the 4.5hrtPA, 6hrtPA, A + 6hrtPA, NA + 6hrtPA, LPS + 6hrtPA, and DNase I + 6hrtPA groups received an intravenous injection of rtPA (10 mg/kg) through the tail vein at the designated time following the establishment of model.

### 2.6 Inhibitor and agonist intervention

Rats treated with LPS + 6hrtPA and DNase I + 6hrtPA were injected with LPS (1.8 mg/kg) and DNase I (2.5 mg/kg) via the tail vein 1 h before modeling.

### 2.7 Euthanasia methods for animals

At 24 h after modeling, rats were euthanized via intraperitoneal injection of 3% sodium pentobarbital aqueous solution at a dose of 120 mg/kg through overdose anesthesia.

### 2.8 Laser speckle imaging

Regional cerebral blood flow (rCBF) alterations were assessed utilizing a laser speckle imaging system (RFLSIIII, RWD Life Technology Co.) at various time points: prior to modeling, immediately following modeling, and 24 h post-modeling. The skulls of the rats were exposed to facilitate the creation of cranial windows and to reduce the thickness of the skull bone prior to imaging. Subsequently, saline was applied to the closed cranial window and imaged using instrumentation.

### 2.9 Measurement of neurological function

Following a 24 h postoperative period, an evaluation of neurological function was conducted using the modified Bederson et al. clinical scale: 0 indicates no observable neurological deficit; 1 signifies contralateral forelimb flexion; 2 reflects a diminished grip in the contralateral forelimb when the tail is pulled; 3 denotes spontaneous movement in all directions, with contralateral circling occurring only when the tail is pulled; and 4 represents spontaneous contralateral circling; 5, Death. Neurological function was assessed using the Garcia JH score, which is evaluated on a scale ranging from 0 to 18, where a higher score signifies a lower degree of neurological impairment. Grip test assessment in rats used suspended metal wires. The rats were subsequently scored based on their performance as follows: 0 for falling off the wire; 1 for hanging onto the wire with one or both forepaws; 2 for hanging with one or both forepaws while attempting to climb onto the wire; 3 for hanging with one or both forepaws in addition to one or both hind paws; 4 for hanging with both fore and hind paws while also wrapping the tail around the wire; and 5 for successfully escaping to the supports.

### 2.10 Infarct volume measurement

Rats were anesthetized 24 h later after modeling, and decapitated them to remove the brain, then sliced the brain into 2 mm thick sections. The brain was subsequently sectioned into 2 mm thick slices. These slices were then incubated in a 2% TTC solution (Sigma) at a temperature of 37°C for 15 min. The images obtained from the brain slices were analyzed utilizing ImageJ software. The calculation of the volume of infarcted regions was performed utilizing the following formula: infarcted brain volume (%) = (total brain volume − stained brain volume)/total brain volume × 100%.

### 2.11 H&E staining

24 h after cerebral infarction, anaesthetized rats received cardiac perfusion of saline followed by perfusion of 4% paraformaldehyde The brain tissues were subjected to fixation in a 10% formalin solution for a period ranging from 24 to 48 h, and then embedded in paraffin. The paraffin-embedded tissues were sectioned into slices (6 μm). Hematoxylin and eosin (HE) staining was performed on the deparaffinized sections. Upon completion of the staining process, subsequently sealed and finally observed under a light microscope (Olympus, Japan) at 200x magnification.

### 2.12 Measurement of blood–brain barrier permeability

Rats that had undergone a stroke were given a 2% solution of Evans blue (EB) dye (Sigma Aldrich, St. Louis, MO, United States) (0.4 mL /100 g) through the tail vein, 2 h prior to euthanasia. Rapid removal of the right brain after cardiac perfusion with saline. Each 100 mg of brain tissue was homogenised in 500 μL formamide, followed by a water bath (24 h, 60°C) and subsequent centrifugation (4°C, 10,000 × g, 20 min). The absorbance of EB in the supernatant was measured at a wavelength of 620 nm utilizing a spectrophotometer. The concentration of EB in micrograms per gram (μg/g) was calculated utilizing the formula: EB concentration (μg/mL) multiplied by the volume of formamide (mL) divided by the mass of brain tissue (g).

The permeability of the blood–brain barrier (BBB) is also assessed through small animal *in vivo* imaging. Cardiac perfusion is performed to remove the whole brain, which is then placed in a small animal imaging system (Digital Precision Medicine) for imaging (excitation wavelength 450–470 nm, emission wavelength 530 nm) to observe the EB fluorescence expression in the brain.

### 2.13 Transmission electron microscopy

Rats were anaesthetized with saline cardiac perfusion, brain tissue was then quickly sectioned into 1 mm^3^ cubes, which were immersed in a 2.5% glutaraldehyde solution at 4°C for the entire night. After being washed with PBS, tissue blocks were fixed in a 1% osmium tetroxide solution for 2 hours, followed by dehydration. Following a 20 min exposure to pure acetone, the tissues were embedded and left to incubate at 70°C overnight. Using a Reichert ultramicrotome, ultrathin sections of 80 nm were created, followed by staining the tissue with 3% uranyl acetate and lead citrate. Transmission electron microscopy (TEM) was employed to assess the ultrastructural alterations of the BBB.

### 2.14 Measurement of hemorrhagic transformation

The right side of the rat brain tissue was taken after perfusion. After homogenizing the brain tissues in 2 mL of PBS, the mixture was centrifuged at 13,000 rpm for 30 min, and the supernatant was collected for further study. A haemoglobin assay kit (QuantiChromTM, Holland, OH, United States) was used to measure haemoglobin concentrations, and a microplate reader was employed to assess the optical density.

### 2.15 Measurement of brain water content

The right cerebral hemisphere wet weight was weighed after removing the brain. Subsequently, it is placed in an oven at 100°C, and after 24 h, the dry weight is determined, which allows for calculations. The formula is: Brain water content = (wet weight − dry weight) / wet weight × 100%.

### 2.16 Western blot analysis

Quantification of total right brain proteins using BCA assay kit (P0010, Biyuntian Biotechnology, China). Proteins in equal amounts were separated using 8–12% SDS-PAGE gels and then transferred to PVDF membranes (Millipore, United States). Overnight incubation of the membranes at 4°C was performed with primary antibodies against anti-MPO (1:4000, Proteintech, 22225-1-AP), anti-NE (1:1000, Abcam, AB314916), anti-Cit H3 (1:1000, Abcam, AB281584) and GAPDH (1:10000, Proteintech, 10494-1-AP). Membranes were then incubated with peroxidase-conjugated goat anti-rabbit IgG (H + L) or goat anti-mouse IgG (H + L) (1:10000, Yeasen, China) for 1 h at room temperature. Chemiluminescence detection was subsequently conducted using ECL reagents (36208ES76, Yeasen, China) in conjunction with a Fusion Edge Multi-function Imaging System (Vilber, France). ImageJ software was used to analyse the relative optical densities of the protein bands.

### 2.17 Real-time polymerase chain reaction

Total RNA was extracted using TRIzol reagent (10606ES60, Yeasen, China). Subsequently, total RNA was reverse transcribed into cDNA using the Hifair^®^ III 1st Strand cDNA Synthesis SuperMix kit (11141ES60, Yeasen, China). Quantitative PCR analysis was then performed on the ViiATM 7 real-time PCR system (ViiATM 7, Applied Biosystems, CA, United States). The analysis was conducted by employing the 2^−ΔΔCt^ method. The primer sequences utilized for the genes analyzed are as follows: CXCL1-forward ACCCAAACCGA AGTCATAGC, CXCL1-reverse ACTTGGGGACACCCTTTAGC; CXCL2-forward ATCCAGAGCTTGACGGTGAC, CXCL2-reverse AGGTACGATCCAGGCTTCCT; CXCL8-forward CCCCCATGG TTCAGAAGATTG, CXCL8-reverse TTGTCAGAAGCCAGCG TTCAC; MPO-forward GGTCAATCGCAGTGGCTTCAAG, MPO-reverse GCACGCTCCTGGTCCTTGG; NE-forward GGCAG GGATTCACTTCAAGA, NE-reverse GCCATCGGTGCAATCTA TCT; TGF-*β*-forward CGGAGAGCCCTGGATACCACCTA, TGF-β- reverse GCCGCACACAGCAG TTCTTCTCT; GAPDH-forward TGGAATTGTGAGGGAGATG, GAPDH- reverse GCCCAGCAAGGATACT GA.

### 2.18 Immunofluorescence staining

Cardiac perfusion, embedding, sectioning same as 2.11. Antigen repair was performed on the dewaxed slices, and then washed with PBST, subsequently blocked with a solution containing 0.3% Triton X-100 and 5% goat serum in PBS for 60 min at room temperature, followed by primary antibody incubation overnight: rabbit anti-ZO-1 (1:3000, Proteintch, 21773-1-AP), rabbit anti-Occludin (1:100, Santa Cruz, sc-133256), rabbit anti-Claudin 5(1:100, ThermoFisher, 352588) and mouse anti-CD31 (1:100, Invitrogen, MA1-80069). Following this, the sections were washed with PBST and incubated with goat anti-mouse secondary antibody (1:500, Abcam, ab150079) and goat anti-rabbit IgG antibody (1:500, Abcam, ab150077). Finally, an anti-fading medium containing DAPI was used to mount all sections. Fluorescence microscopy (Leica Thunder) was used to analyse the resulting images.

### 2.19 ELISA

Blood samples were obtained from the femoral artery of rats, followed by the separation of serum. Subsequently, add the reagents and serum to the 96-well plate as directed. The concentrations of IL-6, TNF-*α* and IL-1β in serum were then determined using the established standard curve by measuring the absorbance at 450 nm using a microplate reader.

### 2.20 Untargeted metabonomics

100 μL serum of each sample were collected and then mixed with 400 μL precooled methanol. The mixtures were ultrasonically extracted for 20 min after vortexing thoroughly. Proteins were removed after centrifuging at 13000 rpm for 15 min. A centrifugal concentrator was used to concentrate the supernatant. Prior to UPLC-QTOF-MS analysis, samples were redissolved in 100 μL of 50% methanol and filtered through a 0.22 μm membrane. Metabolites were analyzed by ZenoTOF 7,600 system (AB Sciex, United States). The chromatographic separation was performed on a Waters BEH C18 column (2.1 × 50 mm, 1.7 μm). 0.1% formic acid (A) and methanol (B) were used as the mobile phase. The flow rate was set to 0.3 mL/min and the elution program was set up as follows: 1 min, 5% B; 4 min, 95% B; 6 min, 95% B; 9.5 min, 5% B.

### 2.21 Tryptophan-targeted metabonomics

Sample pretreatment and determination was referred to the previous report with a few modifications. The Triple Quad 6,500^+^ system (AB Sciex, United States) was applied for analysis and the chromatographic separation was performed on a Waters BEH C18 column (2.1 × 50 mm, 1.7 μm).

### 2.22 Statistical analysis

Data were analyzed using GraphPad Prism software (version 8.0.2) and expressed as mean ± SEM. Analysis of variance (ANOVA) was used for data showing normal distribution and homogeneity of variance. In contrast, the non-parametric Mann–Whitney U test was used to assess data that did not conform to a normal distribution. The statistical significance was indicated by a value of *p* < 0.05.

## 3 Results

### 3.1 Synergistic power of acupuncture and ultra-time window thrombolysis in reducing cerebral infarction and neurological deficit in MCAO rats

**Figure 1:**
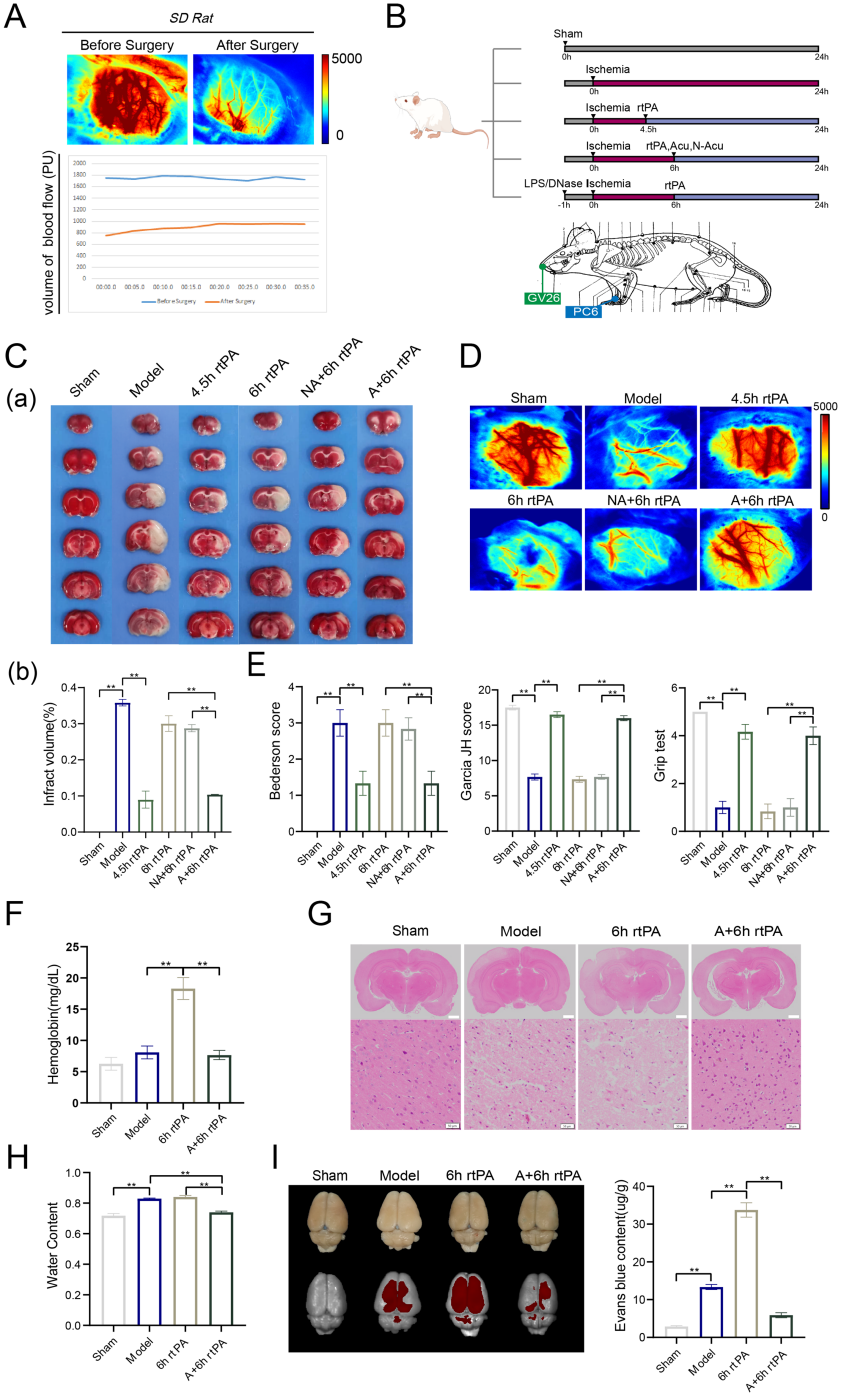
Acupuncture reduced brain injury and reshaped the vascular barrier in ultra-time window thrombolysis. **(A)** Diagram of experimental design. **(B)** Schematic diagram of cerebral blood flow before and after modeling. **(C)** (a) Representative photographs of TTC-stained brain sections in each group. (b) Comparison of cerebral infarct volumes among different groups (*n* = 3). **(D)** Representative images of cerebral blood flow in each group observed using laser speckle imaging 24 h after the establishment of model (*n* = 3). **(E)** Three neurological scores were performed at 24 h after the establishment of model, including bederson test, Garcia JH test and Grip test (*n* = 6). **(F)** Haemoglobin levels in the brain were measured 24 h after modeling. (*n* = 6). **(G)** Representative image of each group’s H&E staining. Scale bars for the images above = 2 mm and below = 50 μm, (*n* = 3). **(H)** Brain water content was observed 24 h after modeling (*n* = 6). **(I)** Representative fluorescent images of EB staining in the brain and EB concentration 24 h after modeling (*n* = 6). All data of results were presented as mean ± SEM, * *p* < 0.05, ** *p* < 0.01.

This study used a rat embolic stroke model, a type of Middle Cerebral Artery Occlusion (MCAO), to recapitulate human ischemic stroke pathophysiology. The model’s success was validated through laser scatter imaging (Figure 1A). Thrombolysis was tested at two time points: within the therapeutic window (4.5 h) as a positive control, and at 6 h as a treatment group (Figure 1B).

TTC staining and laser speckle contrast imaging revealed that the MCAO group exhibited significantly larger cerebral infarct volumes (Figure 1C) and markedly diminished cerebral blood flow (Figure 1D) at 24 h after ischemia compared to the Sham group. Neurological deficits were significant in the model group, as assessed by the Bederson score, Garcia JH score, and grip test (Figure 1E). Administering rt-PA thrombolysis at 10 mg/kg after 4.5 h significantly reduced cerebral infarction and neurological deficits, while ultra-time window rt-PA was mostly ineffective. These findings match clinical observations. Notably, combining thrombolysis with XNKQ acupuncture at 6 h yielded similar benefits to thrombolysis at 4.5 h, including reduced infarct volume, restored perfusion, and improved neurological function. This suggests acupuncture might extend the safe window for rt-PA thrombolysis in ischemic stroke to 6 h. The non-acupuncture point group did not effectively reduce cerebral infarction or neurological deficits, leading researchers to focus on XNKQ acupuncture.

### 3.2 Acupuncture alleviated ICH and cerebral edema caused by ultra-time window thrombolysis via remodeling of vascular barrier

The main adverse effects of ultra-time window thrombolysis are cerebral edema and ICH, limiting rt-PA therapy eligibility. ICH occurrence was assessed by measuring haemoglobin levels in brain tissue homogenates (Figure 1F). The model and sham-operated groups had similar haemoglobin levels, but the 6 h thrombolysis group showed a significant increase, which acupuncture treatment reversed. The location and number of haemorrhages were clearly discernible from e histopathological images of brain tissue sections (Figure 1G). Images from the model group and the ultra-time window thrombolysis group showed evidence of severe cell death and fragmentation, whereas cell fragmentation was significantly ameliorated by combined acupuncture. Furthermore, the model and thrombolysis groups exhibited augmented brain water content, whereas the acupuncture treatment resulted in a reduction of this parameter (Figure 1H). These findings indicate that acupuncture may mitigate adverse effects associated with ultra-time window thrombolysis.

With advancing age, vascular elasticity diminishes and vessel wall integrity declines, resulting in heightened permeability. In ischemic stroke thrombolysis, this can cause more water and blood to leak from vessels, resulting in cerebral edema and ICH. Evans blue (EB), a fluorescent dye, is used to measure vascular permeability. In the study, EB was injected into rats, and its penetration in the cerebral cortex was measured using imaging. The model group showed significant cerebrovascular leakage, worsened by 6 h rt-PA thrombolysis. However, combining acupuncture with thrombolysis significantly reduced fluorescence intensity. EB absorbance in brain tissue was measured at 620 nm, confirming the imaging results (Figure 1I). The results demonstrated that acupuncture exerted protective effects against cerebral edema and hemorrhagic transformation in MCAO rats, potentially through reducing vascular permeability.

### 3.3 Tryptophan and its indole-metabolic pathway were defined as the major contributors mediating the efficacy of acupuncture

Untargeted metabolomic analysis identified 112 metabolites in positive ion mode and 86 in negative ion mode to elucidate the mechanisms underlying acupuncture-mediated extension of the thrombolytic therapeutic window. After SERRF normalization, principal component analysis (PCA) confirmed the reliability of the analytical method by showing clustering of quality control samples (Supplementary Figure S1A). The PLS-DA model was utilized to conduct pairwise comparisons between the model group and the sham-operated, rtPA, and rtPA+Acu groups, respectively, with the aim of identifying differential metabolites (VIP > 1, *p* < 0.05). Significant differences were found between the sham-operated and model groups, with distinct metabolic profiles (Supplementary Figure S1B). In both negative and positive ion modes, the specific parameters were R2X = 0.829, R2Y = 0.997, Q2 = 0.732 and R2X = 0.844, R2Y = 0.998, Q2Y = 0.699, respectively. A permutation test (*n* = 200) confirmed no overfitting. The model also showed significant differences in metabolic profiles between the model and rtPA groups, and the model and rtPA+Acu groups, supporting the model’s validity (Supplementary Figure S1B).

**Figure 2:**
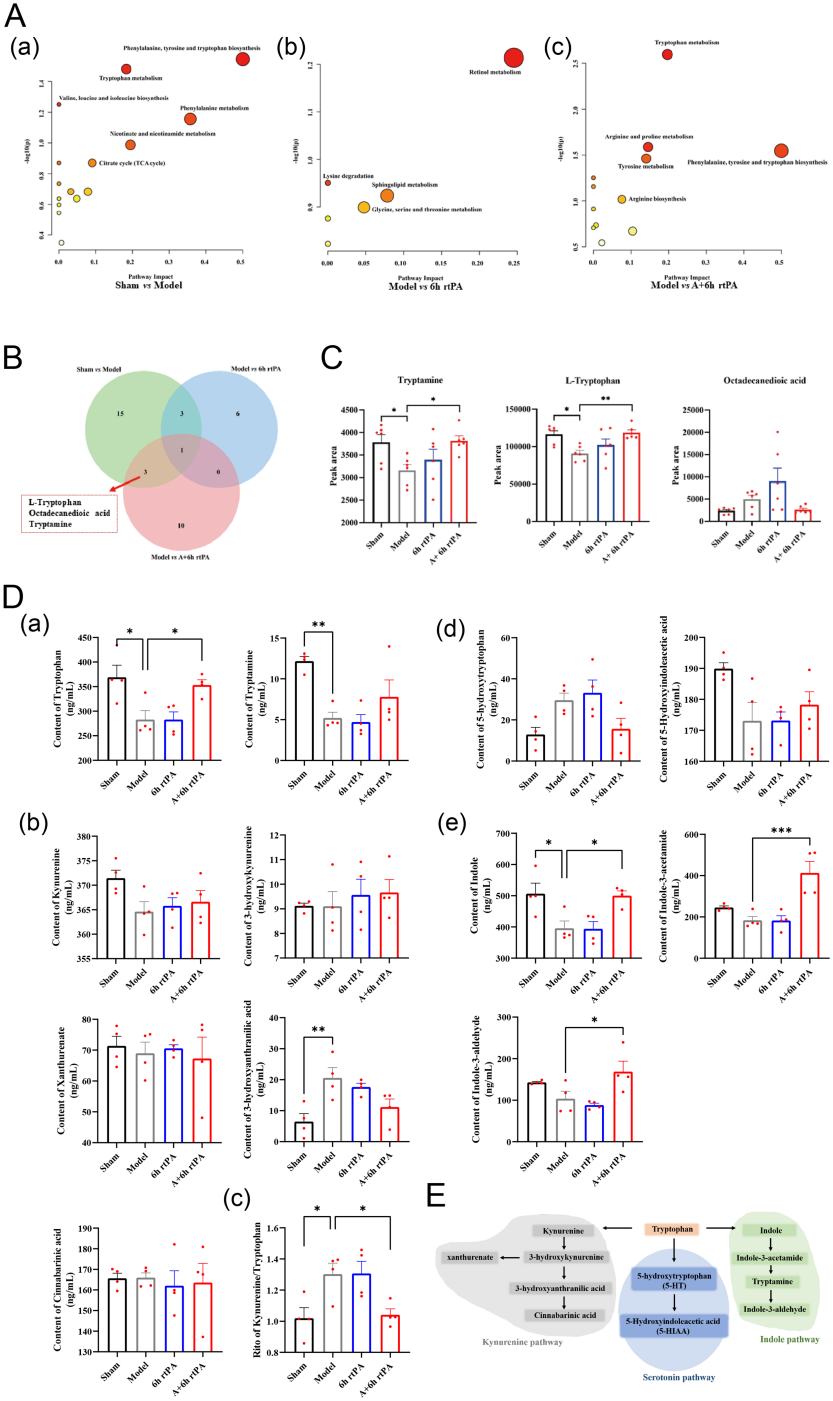
Indole pathway is the key point for the effects of acupuncture. **(A)** Detailed metabolic pathway analysis of differential metabolites. **(B)** Venn diagram of differential metabolites. **(C)** Relative contents of three differential metabolites that specially regulated by acupuncture (*n* = 6). **(D)** Quantitative analysis of metabolites involved in tryptophan metabolism (*n* = 4). **(E)** Schematic representation of tryptophan metabolism. All data of results were presented as mean ± SEM, * *p* < 0.05, ** *p* < 0.01, *** *p* < 0.001.

In total, 22 differential metabolites were identified between the Model and Sham groups, 10 between Model and rtPA, and 14 between Model and rtPA+Acu. Metabolites were analyzed via MetaboAnalyst, revealing significant metabolic pathway changes in the stroke group compared to controls, affecting pathways like phenylalanine, tyrosine, and tryptophan biosynthesis (Figures 2Aa). A Venn diagram analysis showed that adding acupuncture specifically modulated tryptophan, tryptamine, and octadecanedioic acid compared to the 6 h thrombolysis group (Figures 2Ab). The 6 h thrombolytic treatment combined with acupuncture significantly altered tryptophan, arginine, proline, phenylalanine, tyrosine, and related metabolic pathways (Figures 2Ac). A Venn diagram showed that acupuncture specifically affected tryptophan, tryptamine, and octadecanedioic acid compared to the 6 h thrombolysis alone (Figure 2B). Cerebral infarction reduced tryptophan and tryptamine levels, but their levels were notably lowered with the combined treatment (Figure 2C). Metabolic pathway analysis suggests that modulation of the tryptophan metabolic pathway may contribute to the extension of the thrombolytic therapeutic window when combined with acupuncture in stroke treatment.

To investigate this hypothesis, we performed targeted metabolomics, confirming tryptophan and tryptamine trends seen in non-targeted analyses (Figures 2Da). Additionally, we examined three distinct metabolic pathways of tryptophan metabolism: the kynurenine pathway, the serotonin pathway, and the aromatic hydrocarbon receptor pathway as reported (20) (Figure 2E). Research shows high tryptophan levels are inversely linked to cardiovascular disease, while kynurenine levels and the kynurenine/tryptophan ratio are positively linked to higher risk. Our findings indicated that acupuncture combined with thrombolysis at 6 h minimally affected the kynurenine pathway (Figures 2Db). But reduced the Kynurenine/Tryptophan ratio, supporting this treatment’s efficacy (21) (Figures 2Dc). Integrating acupuncture with thrombolysis administered 6 h after an event significantly enhanced the indole metabolic pathway, increasing levels of indole, indole-3-acetamide, and indole-3-aldehyde, unlike thrombolysis alone (Figures 2De).

### 3.4 Acupuncture may inhibit NETs degradation and related inflammatory responses via the indole pathway

NETs are crucial innate immune defenses that impact ischemic stroke by promoting thrombosis and worsening neurological damage. In cerebral ischemia/reperfusion injury (CI/RI), NETs can be anti-inflammatory at moderate levels but excessive NET release, or NETosis, exacerbates inflammation, promotes thrombosis, damages the BBB, and causes further neuronal and tissue injury (22). Indole and its metabolites reduce the activity of the NETs-associated protease MPO, thereby suppressing NETs, which may explain the effectiveness of acupuncture and thrombolysis (23, 24).

**Figure 3:**
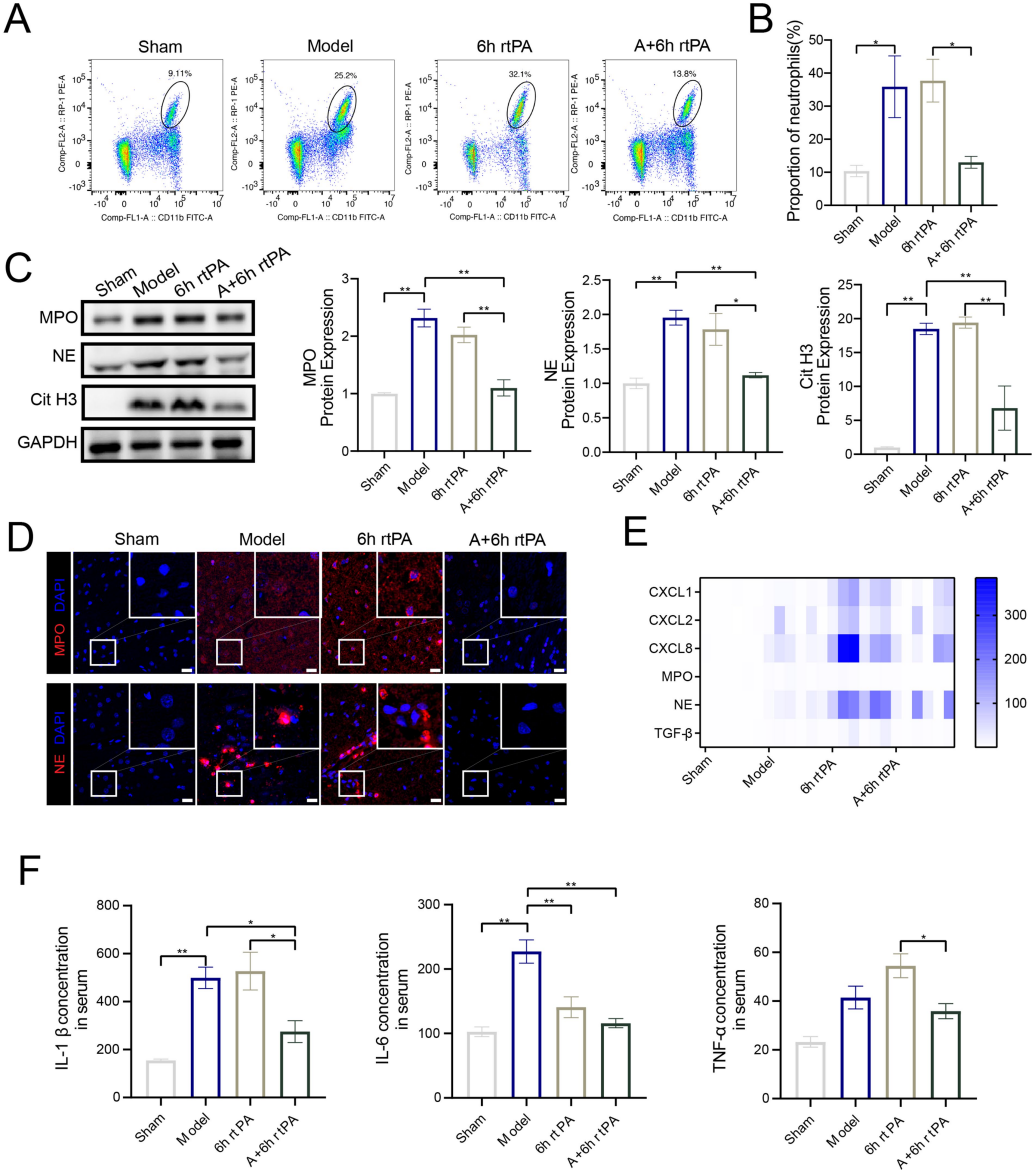
Acupuncture inhibits NETs related proteins and inflammatory response. **(A)** Representative cell flow images of neutrophil count in each group. **(B)** Neutrophil ratio in each group (*n* = 6). **(C)** Western blot and quantitative analysis of MPO, NE and Cit H3 (*n* = 4). **(D)** Representative image of each group’s immunofluorescence staining of MPO and NE. Scale bars for the images = 20 μm. **(E)** Heatmap showing expression pattern of representative genes with NETs (*n* = 6). **(F)** The serum ELISA expression of IL-*β*, IL-6 and TNF-*α* (*n* = 3–5). All data of results were presented as mean ± SEM, * *p* < 0.05, ** *p* < 0.01.

Flow cytometry showed increased peripheral neutrophils in model rats compared to sham-operated ones, with delayed thrombolysis worsening this effect (Figures 3A,B). Acupuncture, however, reduced peripheral neutrophils, likely by lowering neutrophil chemokines like chemokine ligand (CXCL)1, CXCL2, CXCL8 and the neutrophil-activating cytokine transforming growth factor factor-*β* (TGF-β).

**Figure 4:**
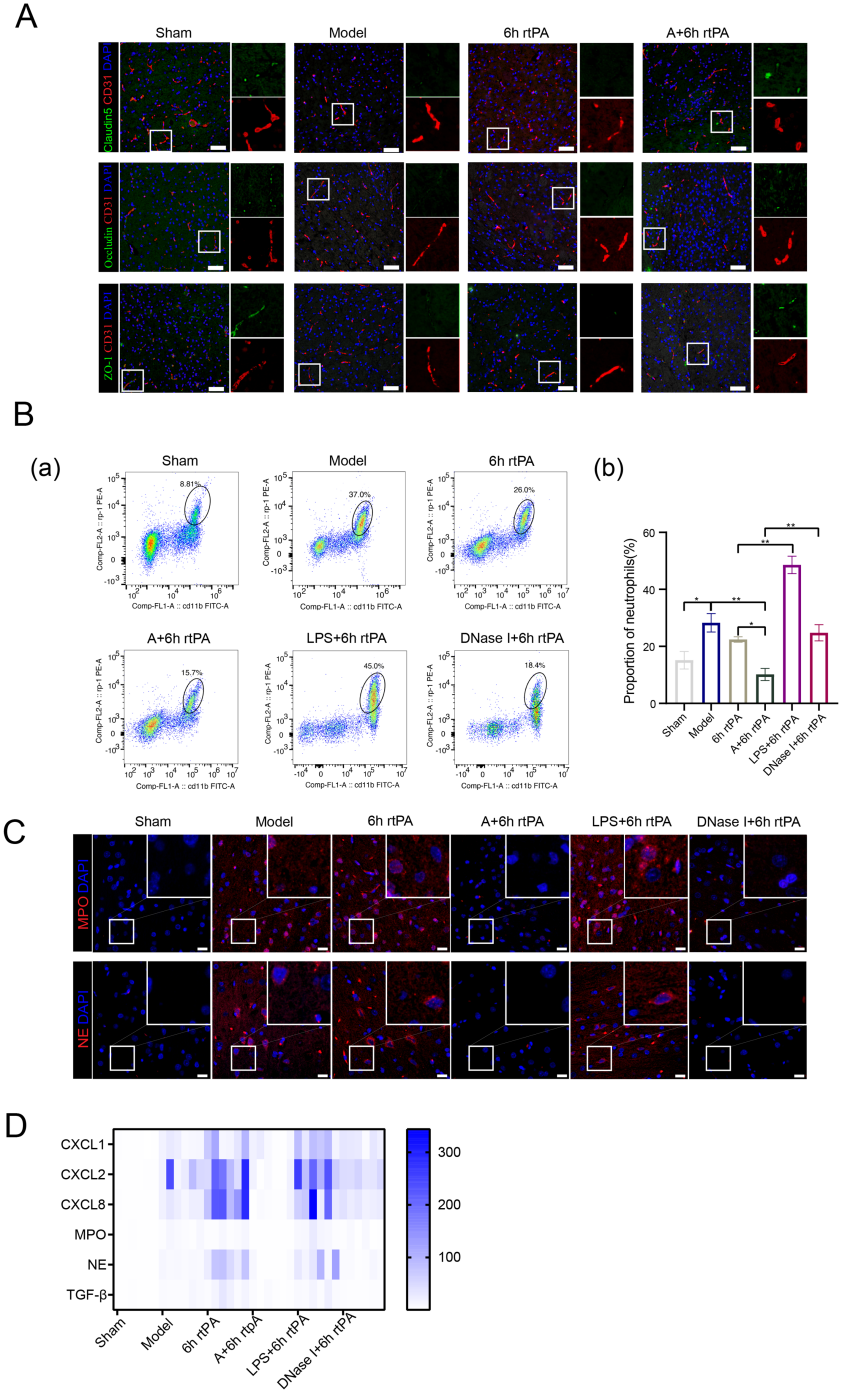
NETs is the key to regulating vascular permeability. **(A)** Representative immunofluorescence images of Claudin5, Occludin and ZO-1 in the IP area of each group, along with a comparison of fluorescence intensity. **(B)** (a) Representative cell flow images of neutrophil count in each group. (b) Neutrophil ratio in each group (*n* = 6). **(C)** Representative image of each group’s immunofluorescence staining of MPO and NE. Scale bars for the images = 20 μm. **(D)** Heatmap showing expression pattern of representative genes with NETs (*n* = 6). All data of results were presented as mean ± SEM, * *p* < 0.05, ** *p* < 0.01.

To assess the impact of ultra-time window rt-PA thrombolysis and acupuncture on NET formation in brain tissue, western blotting, PCR, and immunofluorescence techniques were employed. The findings found a significant increase of NETs-associated proteases, including myeloperoxidase (MPO), Neutrophil elastase (NE), and Citrulline Histone 3 (CitH3), in the brains of model rats (Figures 3C,D). Ultra-time window rt-PA thrombolysis reduced NE levels but did not significantly affect MPO and CitH3 (Figures 4C,D). Notably, combining thrombolysis with acupuncture notably decreased MPO, NE, and CitH3, thereby inhibiting NETs formation. mRNA levels of MPO matched protein expression patterns, with similar but non-significant trends for NE (Figure 3E). ELISA analyses (Figure 3F) revealed a significant increase in the serum concentrations of inflammatory cytokines, specifically Interleukin-1β (IL-1β) and Interleukin-6 (IL-6), in the model group. Ultra-time window rt-PA thrombolysis reduced IL-6 but increased TNF-*α*. The combined thrombolysis-acupuncture therapy significantly suppressed the secretion of these three cytokines, supporting acupuncture’s role in reducing NETs-related inflammation.

### 3.5 Acupuncture reduced vascular permeability in a NETs-dependent manner

NETs contribute to vascular hyperpermeability in cancer pathology by degrading endothelial tight junctions (TJs) through protease release. To explore if a similar mechanism occurs in ischemic stroke, the effect on endothelial TJs was examined using immunofluorescence staining. The study found decreased expression of tight junction proteins like Claudin5, Occludin, and ZO-1 in both the model and 6 h thrombolysis groups (Figure 4A). However, combining acupuncture with thrombolysis significantly enhanced tight junction expression.

### 3.6 Pharmacological modulators of NETs exerted a significant effect on cerebral vascular permeability in MCAO rats

To strengthen the connection between neutrophil NETs and vascular permeability in ischemic stroke, lipopolysaccharide (LPS) was administered to induce NETs formation, while Deoxyribonuclease I (DNase I) acted as a positive control (25). Flow cytometry and immunofluorescence showed that LPS increased peripheral neutrophils and NET production in brain tissue. In contrast, DNase I did not change neutrophil counts but significantly reduced NET-related proteins MPO and NE, as well as NE mRNA levels (Figures 4B,C). DNase I also decreased cytokines CXCL1, CXCL8, and TGF-*β* compared to the 6 h thrombolysis group.

**Figure 5:**
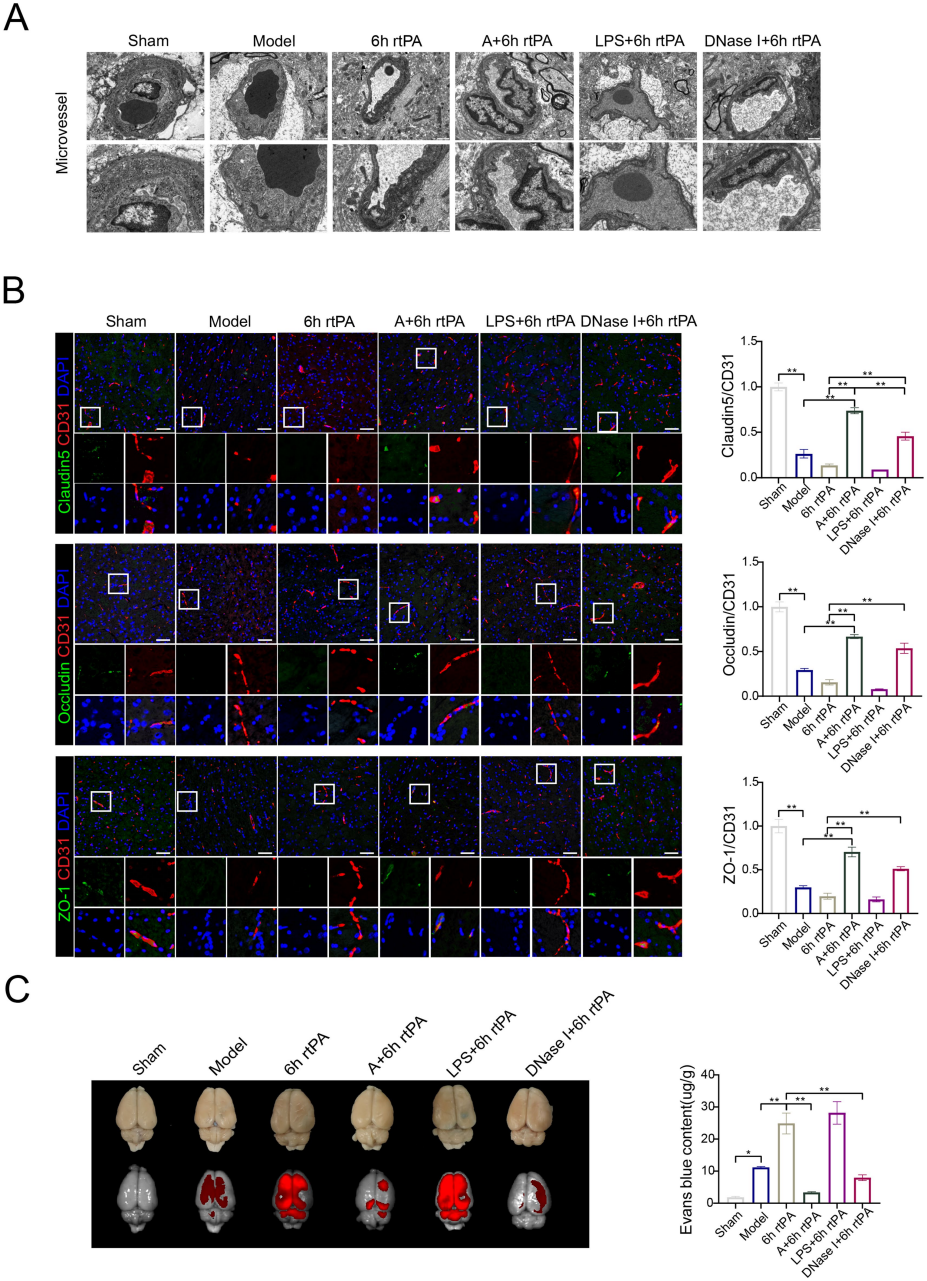
Acupuncture reduces vascular permeability in a NETs-dependent manner. **(A)** Transmission electron microscopy of microvessel (scale bars for the images above = 1 μm and below = 500 nm). **(B)** Representative immunofluorescence images of Claudin5, Occludin and ZO-1 in the IP area of each group, along with a comparison of fluorescence intensity. **(C)** Representative fluorescent images of EB staining in the brain and EB concentration 24 h after modeling (*n* = 6). All data of results were presented as mean ± SEM, * *p* < 0.05, ** *p* < 0.01.

We initially employed electron microscopy to examine brain micro-vessel changes with NET stimulation or inhibition (Figure 5A). In the model group, microvascular abnormalities include dispersed endothelial nuclei, loose vascular endothelium, fewer organelles, swollen mitochondria with reduced or absent cristae, and disrupted TJs. The thrombolysis group also shows abnormal microvascular structure, with dense, swollen, and fractured mitochondria and unclear basement membrane. Acupuncture significantly restores vascular structure, improving endothelial cells and protecting TJs. Administering LPS before modeling worsened mitochondrial swelling in endothelial cells and further disrupted the basement membrane and tight junctions (TJs). However, acupuncture or DNase I effectively reduced vascular damage from ultra-time window thrombolysis, restoring endothelial integrity and preserving TJs. The data suggest that NETs compromise blood vessel integrity.

Immunofluorescence and electron microscopy showed consistent TJ alterations across groups (Figure 5B). *In vivo* imaging (Figure 5C) indicated increased fluorescence in the model, 6 h thrombolysis, and LPS early intervention groups compared to the sham group, while acupuncture or DNase I with thrombolysis significantly reduced fluorescence intensity. This finding was further validated through EB fluorescence quantification of brain tissue homogenates (Figure 5C).

### 3.7 Reduction of NETs generation represented an effective adjuvant therapy for ultra-time window thrombolysis

**Figure 6:**
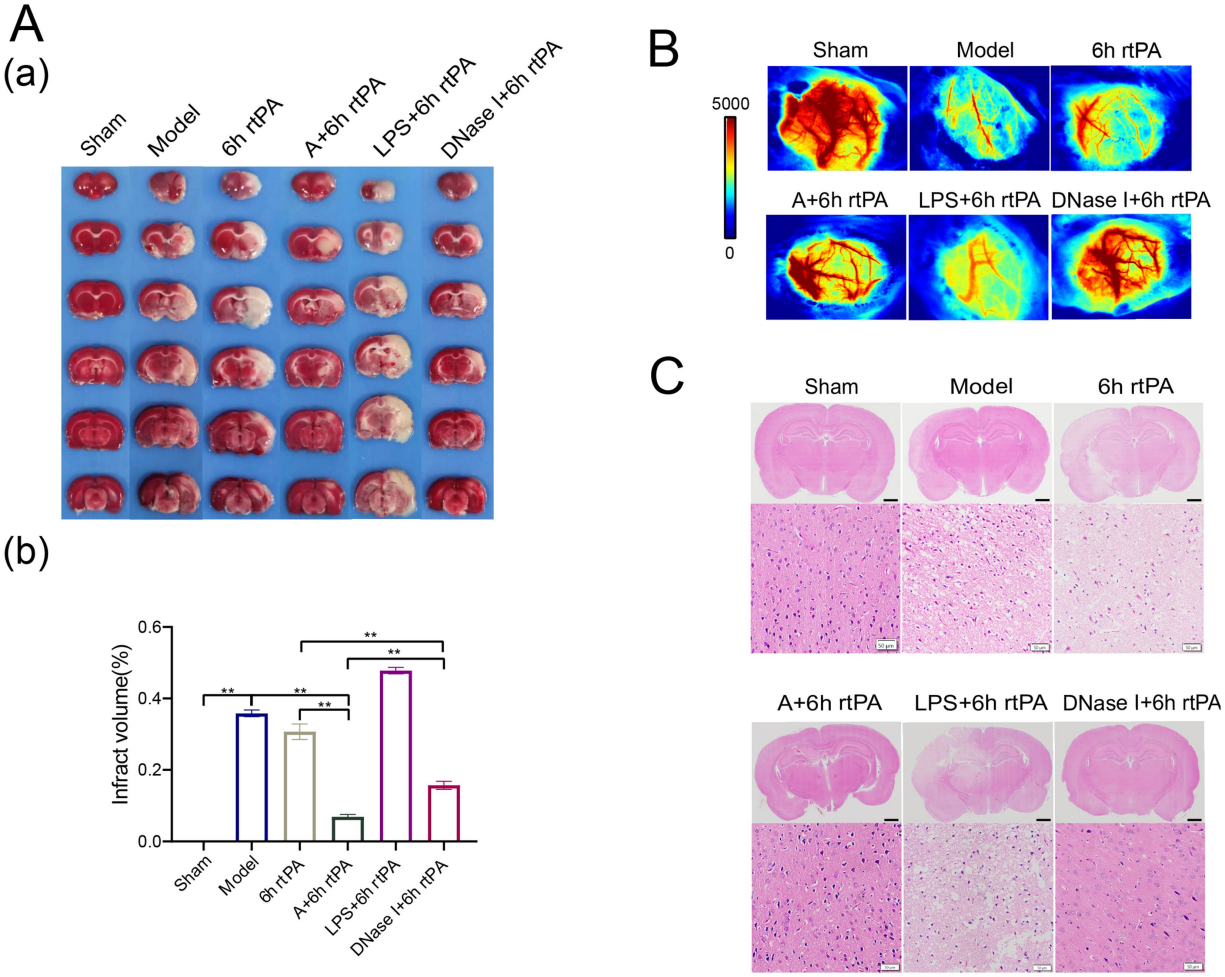
Regulating the generation of NETs can improve the safety of ultra-time window thrombolysis. **(A)** (a) Representative photographs of TTC-stained brain sections in each group. (b) Comparison of cerebral infarct volumes among different groups (*n* = 3). **(B)** Representative images of cerebral blood flow in each group observed using laser speckle imaging 24 h after the establishment of model (*n* = 3). **(C)** Representative image of each group’s H&E staining. Scale bars for the images above = 2 mm and below = 50 μm (*n* = 3). All data of results were presented as mean ± SEM, * *p* < 0.05, ** *p* < 0.01.

Subsequent investigations evaluated the therapeutic potential of NETs modulation in ischemic stroke, based on these findings. The results from TTC staining (Figure 6A) and laser speckle imaging (Figure 6B) showed that increasing NETs via LPS did not significantly impact infarct volume or cerebral perfusion. In contrast, inhibiting NETs with the acupuncture or DNase I significantly reduced infarction. The combination of LPS with thrombolytic therapy was found to exacerbate cellular damage and hemorrhage within brain tissue (Figure 6C). Conversely, DNase I intervention effectively mitigated the pathological damage induced by these factors. These findings suggest that reducing NETs is a promising adjunctive therapy for enhancing ultra-time window thrombolysis in ischemic stroke, improving efficacy, and reducing side effects.

## 4 Conclusion and discussion

Stroke is an acute cerebrovascular disease with two principal types: hemorrhagic and ischemic, the latter accounting for the majority of cases. Intravenous thrombolysis remains the preferred treatment for ischemic stroke effective within 4.5 h of onset. However, most patients with acute ischemic stroke arrive at the hospital more than 4.5 h after the onset of the stroke or the time of onset is not known, resulting in missed thrombolytic opportunities. If a suitable method can be found to extend the safe time window for thrombolysis, the possibility of thrombolysis can be won for numerous patients.

CI/RI injury constitutes the principal etiology of thrombolysis-associated complications beyond the time window. It occurs when blood flow is restored to ischemic tissues, worsening functional and structural damage instead of improving it, potentially causing irreversible harm. A Despite extensive research, the mechanisms of primary and secondary damage remain unclear, possibly involving excessive free radicals, excitatory amino acid toxicity, calcium overload, and vascular dysfunction (26). Neuro cell apoptosis due to vascular dysfunction is crucial in cerebral ischemia–reperfusion injury, suggesting that repairing vascular function during thrombolysis could enhance treatment safety (27). Using electron microscopy and *in vivo* imaging, we assessed the impact of ultra-time window thrombolysis on vascular function. Ultra-time window thrombolysis was demonstrated to induce vascular structural disruption and elevated vascular permeability.

Vascular dysfunction encompasses not only endothelial injury but also intricate crosstalk between immune cells and endothelial cells. During the immune response, the extravasation of immune cells from the bloodstream necessitates a dynamic interaction with endothelial cells, primarily mediated by leukocyte adhesion molecules such as P-selectin, E-selectin, and ICAM-1, in conjunction with their respective receptors, including L-selectin and CD18. These interactions guide immune cells into tissues, crucial for immune responses and linked to diseases like rheumatoid arthritis, autoimmune disorders, and cancer (28, 29). Our findings suggest this pathological process may similarly contribute to ischemic stroke pathogenesis. Neutrophils, the main innate immune cells in the blood, are linked to poor stroke outcomes. Ultra-time window thrombolysis triggers inflammation through NETs, which regulate thrombosis and degrade endothelial proteins. NETs have been documented to regulate thrombosis and degrade endothelial tight junction proteins. Moreover, thrombolysis-induced hemorrhagic transformation has been associated with occludin PKCβ phosphorylation (30). Therefore, the accumulation of NETs may be a critical factor in limiting the efficacy of ultra-time window thrombolysis and inducing adverse effects.

Traditional Chinese Medicine (TCM) offers distinct advantages in the treatment of stroke. Within the framework of Chinese medicine, ischemic stroke attributed to pathological factors such as phlegm and blood stasis, which obstruct the brain orifices. Academician Xuemin Shi, a distinguished figure in national medicine, introduced the therapeutic approach known as XNKQ method. In simplified terms, this concept involves “awakening the mind” This method has demonstrated significant efficacy in stroke treatment. In this study, GV26 and PC6 were selected as the principal acupoints of the XNKQ method. PC6 is a crucial acupoint for regulating mental disorders, functioning to tranquilize the mind and calm the spirit; GV26 serves as an essential acupoint for emergency treatment, with the capacity to restore consciousness and induce resuscitation. As evidenced by our group’s preclinical and animal studies, it is indicated has synergistic power potential on rt-PA, although the underlying mechanisms remain unclear. Contemporary pharmacological research has redefined phlegm as a byproduct of the inflammatory response and equated stasis accumulation with thrombosis, noting a mutually reinforcing relationship between these two factors. Consequently, this study explores the synergistic effects of acupuncture and thrombolysis by examining immune-vascular interactions. The findings reveal that acupuncture inhibits the excessive production of NETs induced by ischemic stroke and thrombolysis, consequently maintaining vascular integrity.

The regulation of NETs is influenced by various factors. To identify upstream regulators, we employed both non-targeted and targeted metabolomics approaches. Non-targeted metabolomics indicated that modulation of tryptophan metabolism might be the primary mechanism by which acupuncture exerts its effects. Tryptophan-targeted metabolomics further identified the tryptophan-indole metabolic pathway as a crucial target for acupuncture. MPO constitutes a critical component of NETs. Research demonstrates that indole and its metabolites suppress MPO activity to reduce NETs levels, and inhibitors of this protease is already in use (31). Based on our findings, it is concluded that acupuncture’s inhibitory effect on NETs recruitment is likely realized through modulation of the tryptophan-indole pathway, thereby preventing vascular leakage, enhancing the efficacy of ultra-time window rt-PA thrombolysis and minimizing side effects.

As previously indicated, our preliminary clinical study demonstrates that combining XNKQ acupuncture with intravenous rt-PA thrombolysis not only enhances neurological functional recovery but also reduces the incidence of ICH post-cerebral infarction. Current clinical research remains confined to the thrombolytic time window due to practical constraints. Ultra-time window rt-PA thrombolysis carries risks of hemorrhagic transformation and cerebral edema—common thrombolytic complications. Our experimental studies confirm that acupuncture mitigates these complications, enhances the safety of ultra-time window rt-PA thrombolysis in cerebral infarction, and extends the therapeutic time window to 6 h. Given acupuncture’s safety, efficacy, and accessibility, widespread clinical implementation of early acupuncture intervention during acute cerebral infarction could effectively overcome limitations of thrombolytic therapy. This approach would enable more patients to receive timely and effective treatment, reducing disability and mortality rates—demonstrating significant clinical utility.

Given the well-documented in the literature regarding the association between nets and thrombosis, and considering that the principal objective of this investigation focuses on safety optimization, thrombus detection was not systematically evaluated. Nonetheless, this study has certain limitations, including the insufficient investigation of neutrophil dynamics. Neutrophils are among the initial cell types to respond to ischemic injury, rapidly accumulating at the lesion site within hours, recruiting additional immune cells, and initiating an inflammatory response that peaks and then diminishes within 3–5 days post-injury (32). Our research concentrated solely on the impact of neutrophils on the vasculature during the acute phase (24 h) and did not examine their effects during the recovery phase. Subsequent investigations will include time points at 3, 5, and 7 days.

Research indicates DNase I has no effect on neutrophil abundance, consistent with our findings (33). Both DNase I and acupuncture reduce NETs, however, DNase I does not decrease neutrophil proportions, whereas acupuncture demonstrates significantly greater efficacy in reducing cerebral infarct volume. We hypothesize that this limitation may partly explain why DNase I has not been adopted clinically for the treatment of cerebral ischemia. Conversely, the sustained efficacy of acupuncture in treating cerebral ischemia may be attributed to its bidirectional immunomodulatory effects (34). Further research is required to elucidate the specific mechanisms involved, with a focus on exploring additional time points.

## Data Availability

The original contributions presented in the study are included in the article/Supplementary material, further inquiries can be directed to the corresponding authors.
