# Supplementary material for: Targeting revascularization via tryptophan-indole-NETs axis: the synergistic power of acupuncture and rt-PA thrombolysis in ischemic stroke
Source: Front Neurol. 2025 Jul 4;16:1596158. doi: 10.3389/fneur.2025.1596158 (PMC12271142; doi:10.3389/fneur.2025.1596158)
Supplement: Supplementary file 1 [file Data_Sheet_1.docx]

Supplementary Material

**
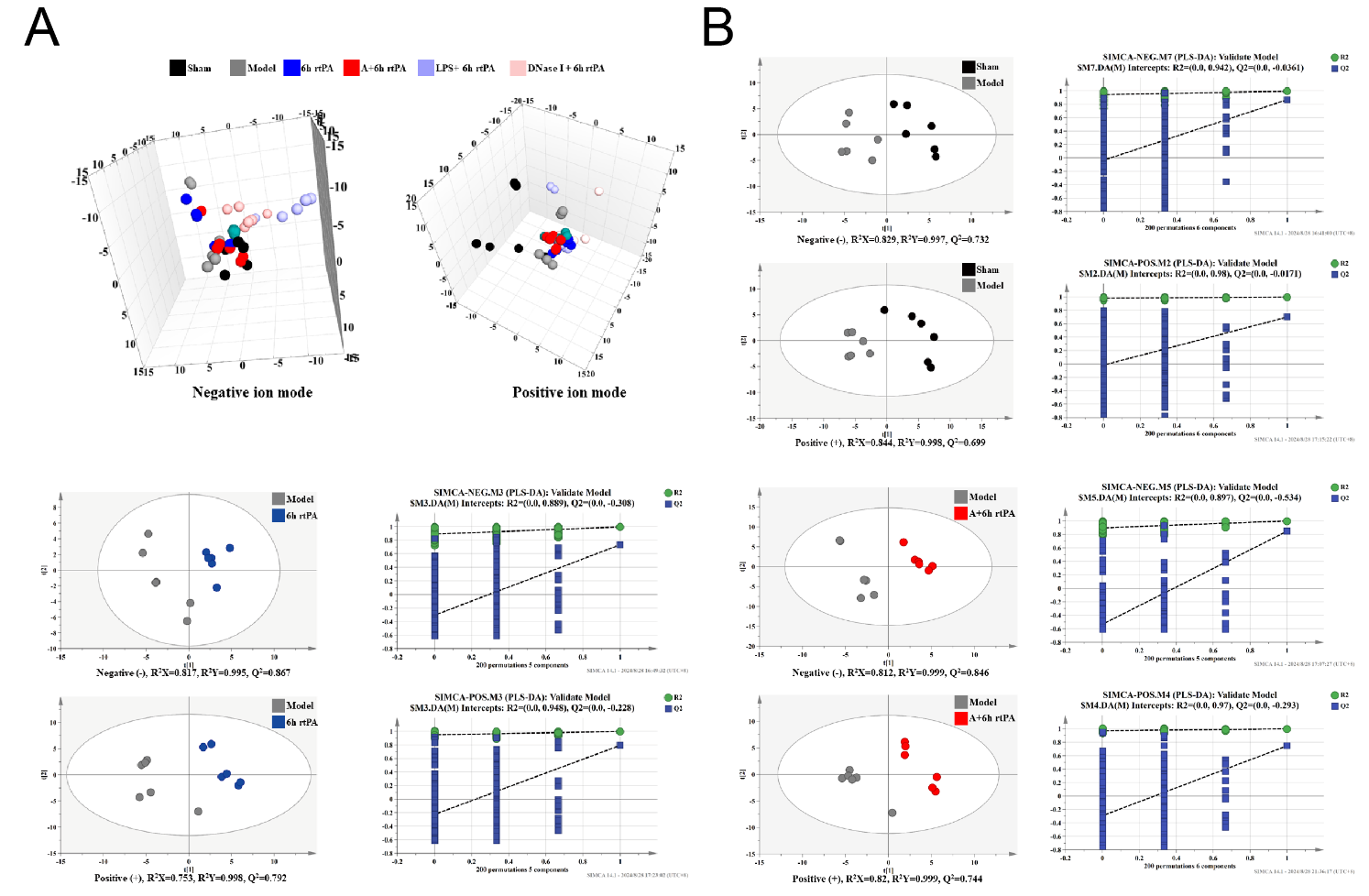
**

**Supplementary Figure 1.** Multivariate data analysis of untargeted metabolomics. (A) PCA plots in negative ion mode and positive ion mode. (B) PLS-DA plots and permutation tests (n = 200) in negative ion mode and positive ion mode.
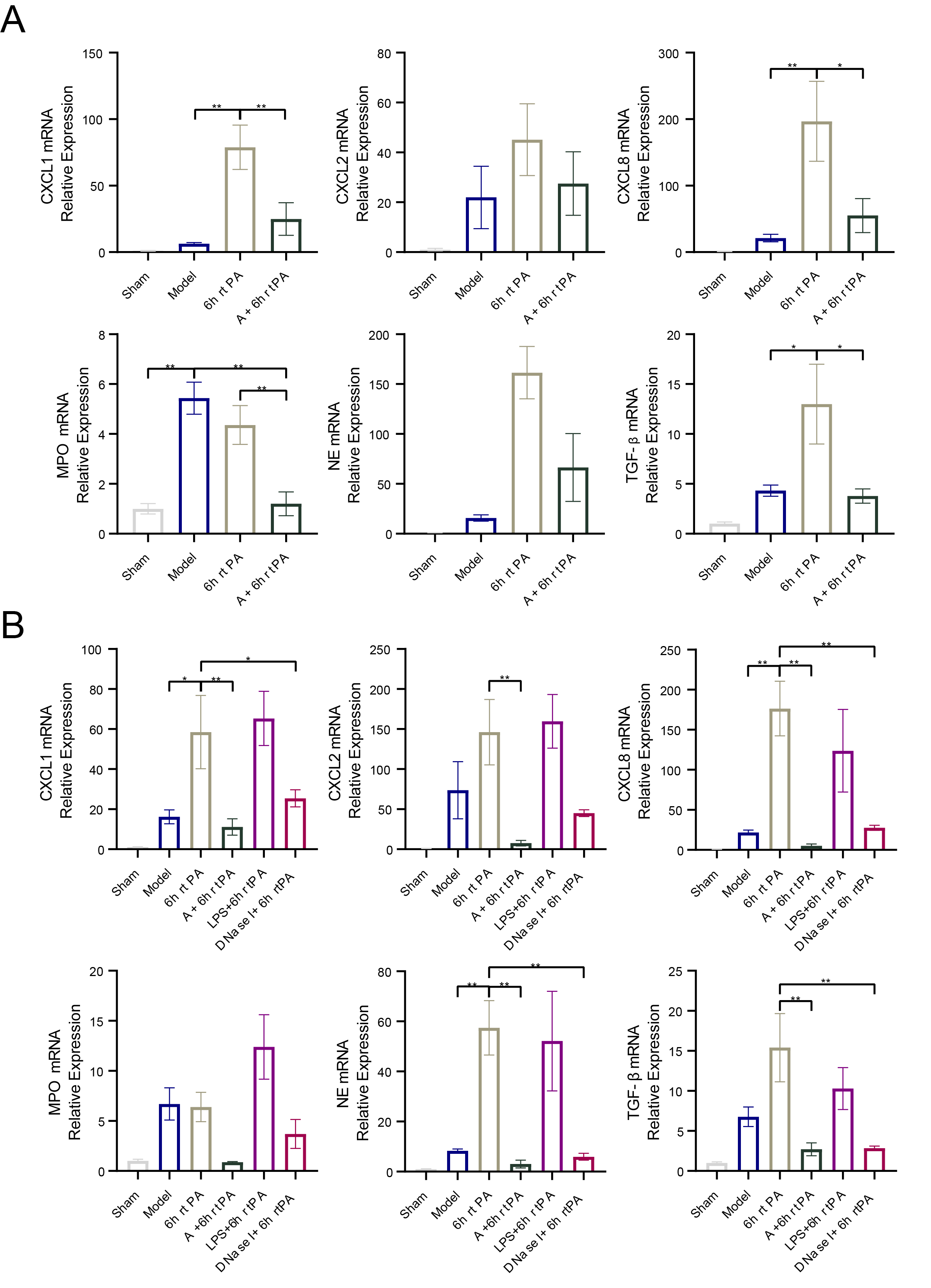


**Supplementary Figure 2.** The effect of acupuncture on the expression of neutrophil-related genes. (A)(B) Expression of NETs related genes in each group (n = 6). All data of results were presented as mean ± SEM, * p < 0.05, ** p < 0.01.


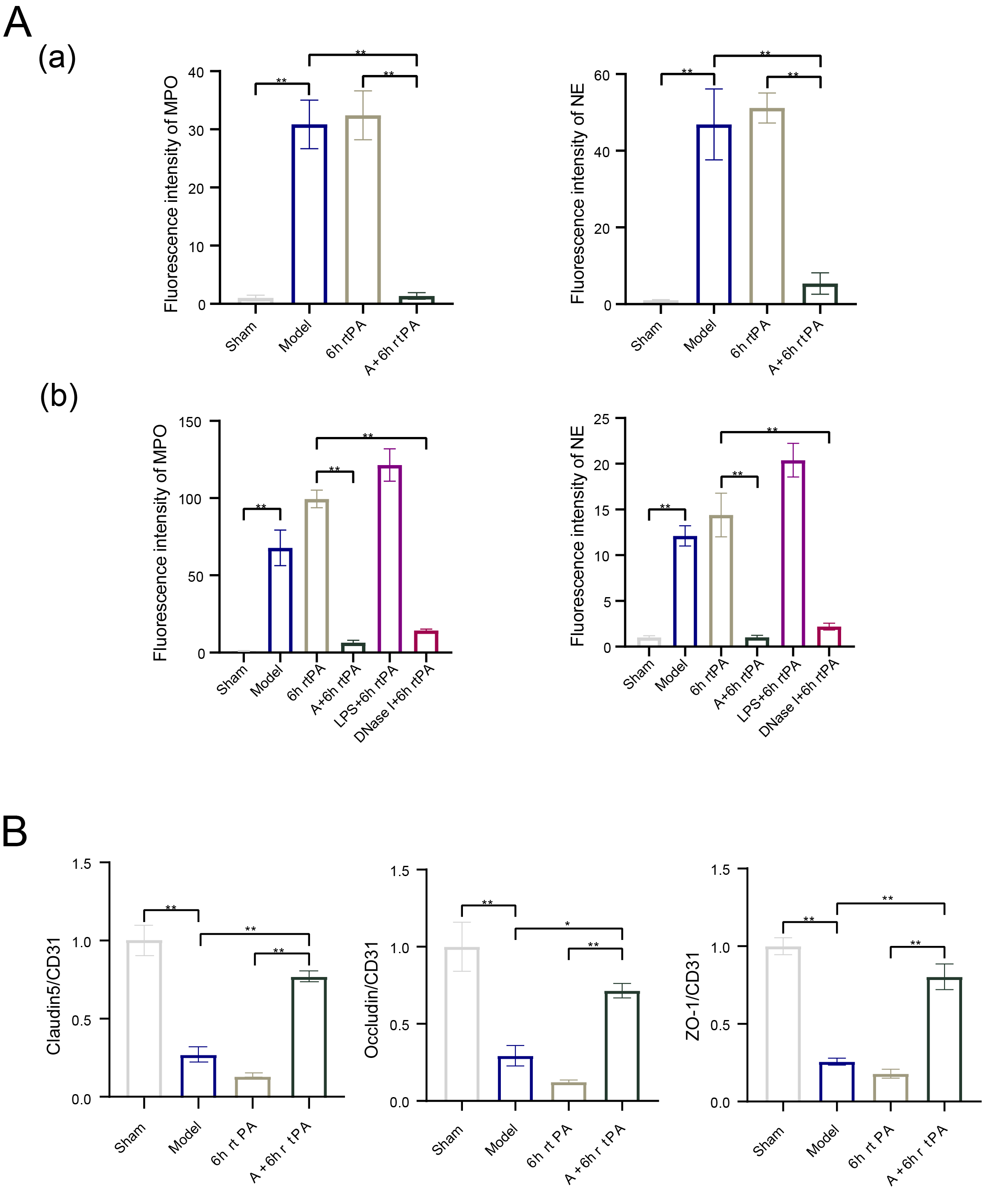


**Supplementary Figure 3.** The effect of acupuncture on the expression of NETs-related proteins and tight junction associated protein. (A)Fluorescence expression of MPO and NE in each group (n = 3). (B) Fluorescence expression of Claudin5, Occludin and ZO-1 in each group (n = 3). All data of results were presented as mean ± SEM, * p < 0.05, ** p < 0.01.


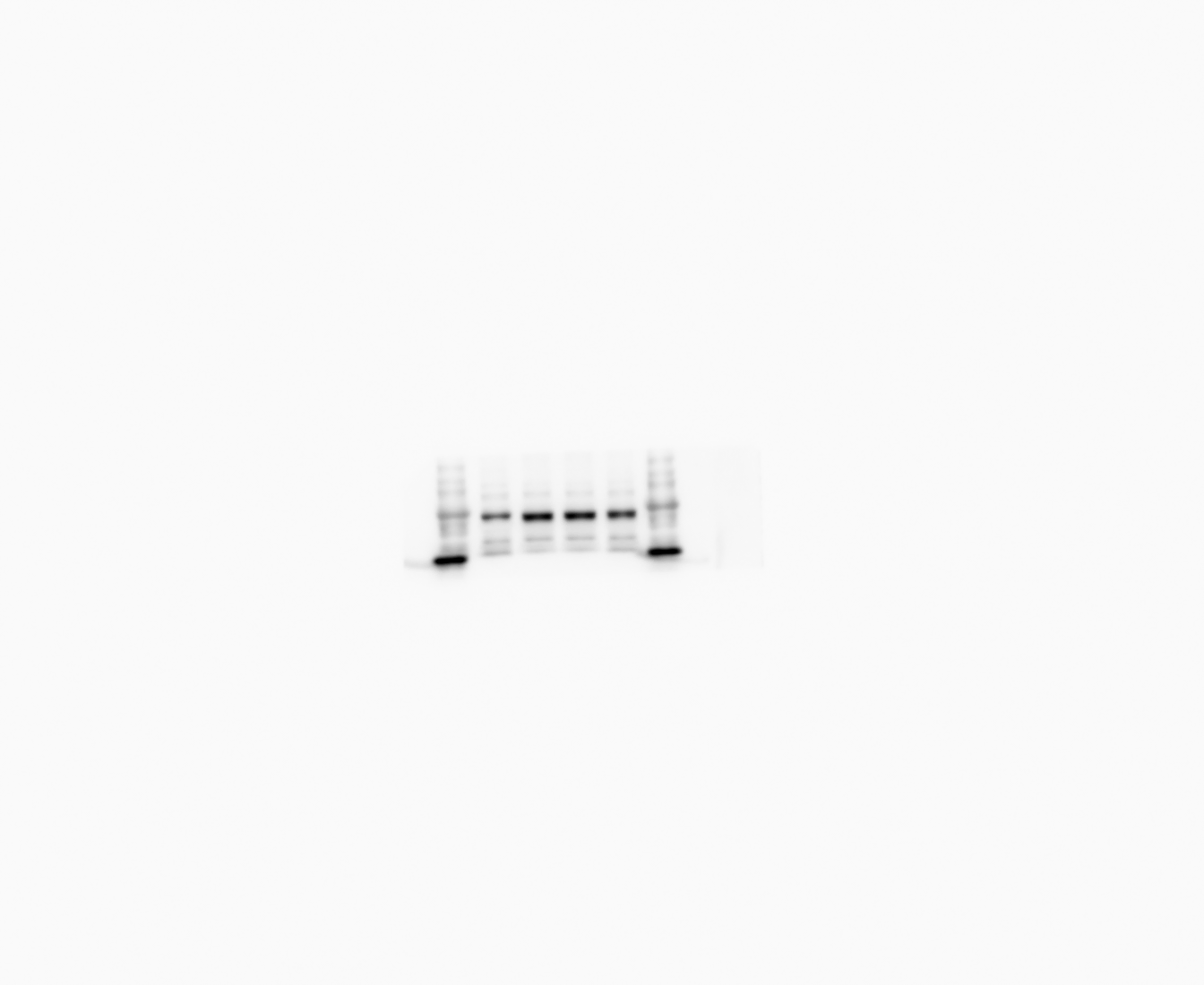


**Supplementary Figure 4.** Original Western blot images of MPO，Groups from left to right are：Sham, Model, 6h rtPA, A+6h rtPA.


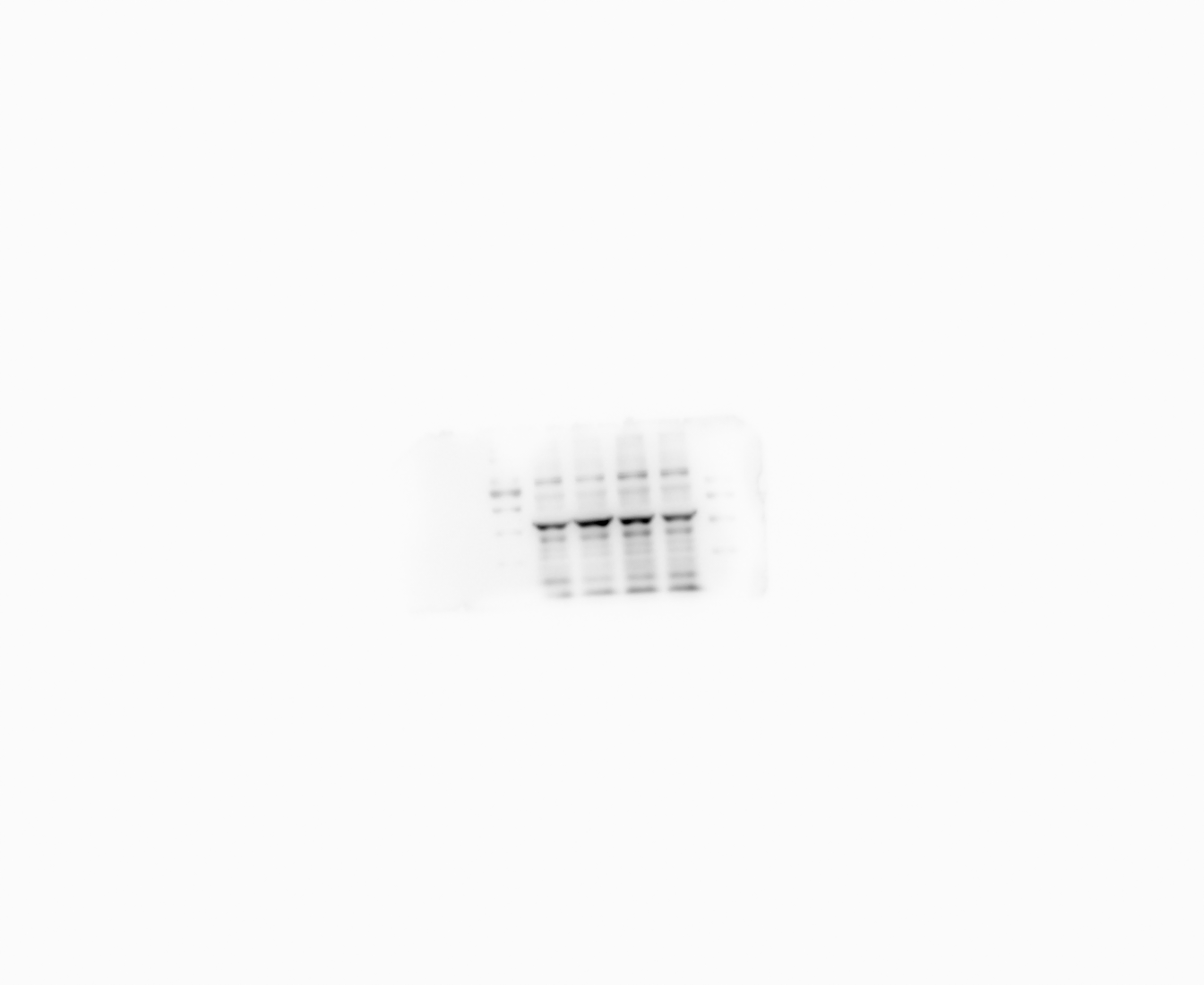


**Supplementary Figure 5.** Original Western blot images of NE，Groups from left to right are：Sham, Model, 6h rtPA, A+6h rtPA.


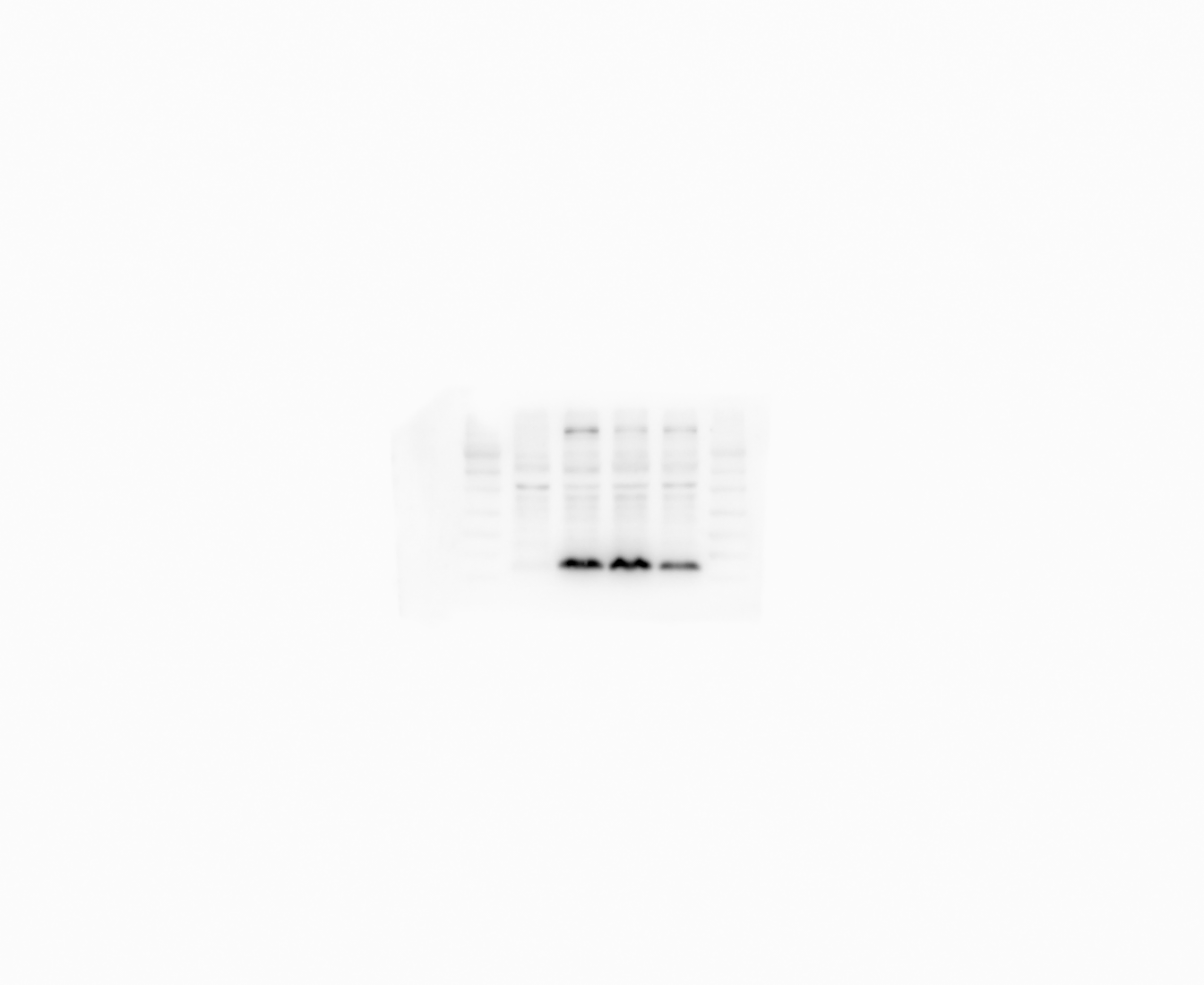


**Supplementary Figure 6.** Original Western blot images of Cit H3，Groups from left to right are：Sham, Model, 6h rtPA, A+6h rtPA.


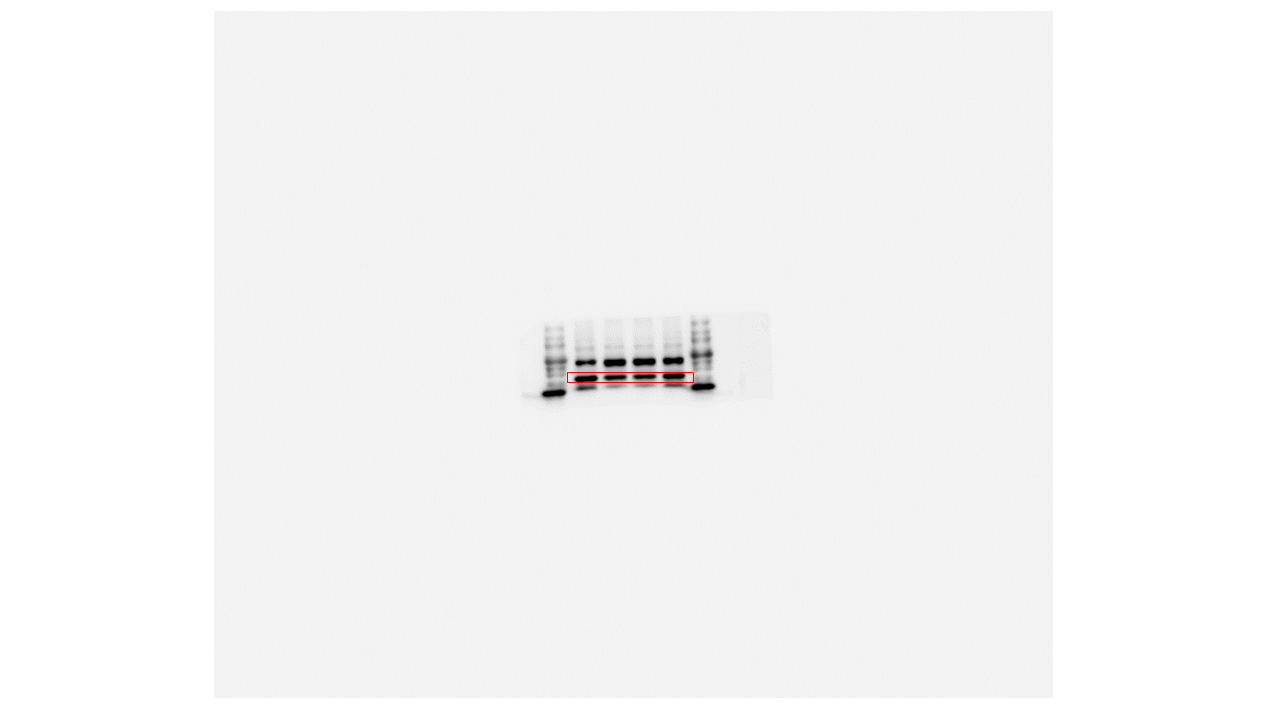


**Supplementary Figure 7.** Original Western blot images of GAPDH (within the red box on the figure)， Groups from left to right are：Sham, Model, 6h rtPA, A+6h rtPA.
